# Molecular and Structural Alterations of Skeletal Muscle Tissue Nuclei during Aging

**DOI:** 10.3390/ijms25031833

**Published:** 2024-02-02

**Authors:** Barbara Cisterna, Manuela Malatesta

**Affiliations:** Department of Neurosciences, Biomedicine and Movement Sciences, University of Verona, Strada Le Grazie 8, 37134 Verona, Italy; barbara.cisterna@univr.it

**Keywords:** cell nucleus, chromatin, gene expression, ribonucleic acid (RNA) transcription, RNA processing, myofiber, satellite cell, skeletal muscle atrophy

## Abstract

Aging is accompanied by a progressive loss of skeletal muscle mass and strength. The mechanisms underlying this phenomenon are certainly multifactorial and still remain to be fully elucidated. Changes in the cell nucleus structure and function have been considered among the possible contributing causes. This review offers an overview of the current knowledge on skeletal muscle nuclei in aging, focusing on the impairment of nuclear pathways potentially involved in age-related muscle decline. In skeletal muscle two types of cells are present: fiber cells, constituting the contractile muscle mass and containing hundreds of myonuclei, and the satellite cells, i.e., the myogenic mononuclear stem cells occurring at the periphery of the fibers and responsible for muscle growth and repair. Research conducted on different experimental models and with different methodological approaches demonstrated that both the myonuclei and satellite cell nuclei of aged skeletal muscles undergo several structural and molecular alterations, affecting chromatin organization, gene expression, and transcriptional and post-transcriptional activities. These alterations play a key role in the impairment of muscle fiber homeostasis and regeneration, thus contributing to the age-related decrease in skeletal muscle mass and function.

## 1. Introduction

Aging severely affects skeletal muscle, causing a progressive decline in muscle mass and a parallel decrease in strength and endurance. This phenomenon affects even healthy, physically active subjects, and the rate of muscle loss in humans has been estimated to range between 1% and 8% per year starting at the age of 30-50 [1,2,3]. This condition, also known as sarcopenia (e.g., [4,5]), is associated with decreased functional performance, a higher risk of falls, and compromised motor function as well as with a number of metabolic and physiological impairments [5,6,7,8,9]. Consequently, sarcopenia represents an important risk factor for frailty, loss of independence, physical disability, and premature death in the elderly. Understanding the mechanisms underlying age-related skeletal muscle wasting and weakness is therefore of primary importance not only to set up efficient therapeutic approaches but also to face a serious healthcare and social problem.

These mechanisms are certainly multifactorial and still remain to be fully elucidated.

Skeletal muscle is a complex and heterogeneous organ. The main component is represented by multinucleated fibers, which are the active element of movement, with their metabolic and functional properties differing between the several fiber types [10,11,12]. In addition to multinucleated fibers, muscle tissue is characterized by mononucleated postnatal myogenic stem cells (satellite cells). Besides these mononucleated cells, other non-muscle mononucleated cell populations, such as connective tissue cells (e.g., fibroblasts), immune cells, vessel-associated cells (e.g., endothelial cells), glial cells (e.g., Schwann cells), and non-myogenic mesenchymal progenitors (e.g., fibro-adipogenic progenitors (FAPs)) [13,14], constitute the substrate capable of influencing both muscle fibers and satellite cells, thus playing a key role in maintaining skeletal muscle homeostasis. Given the complex and fine interactions occurring among these different cell types, it is likely that alterations/modifications of non-muscle cells are involved in the multifactorial process of skeletal muscle aging.

Several hypotheses have been raised, pointing to muscle tissue factors such as the loss of protein homeostasis, along with the impairment of proteolytic and autophagic pathways [15,16,17,18], mitochondrial dysfunction [19,20], the depletion of myofiber nuclei [21,22,23], a reduced number of satellite cells and/or the alteration of their proliferation and differentiation potential [23,24,25,26], myofiber denervation [27,28], environmental factors such as a decrement in microvascular function and exercise tolerance [29,30], an alteration in the hormonal milieu with impaired anabolic signaling [31,32], and increased concentrations of inflammation mediators [33,34,35]. 

The functionality of the cell nucleus has been also considered as a possible contributing factor to sarcopenia. In this cell compartment, DNA (deoxyribonucleic acid) is spatially organized, genes are transcribed, and primary RNA transcripts undergo modifications beforehand to be exported to the cytoplasm [36,37]; therefore, any dysfunction in the nuclear organization and molecular processing may affect muscle structure and function. Interestingly, chromatin dysregulation [38], ribosomal (r)DNA genome instability [39], and reduced/impaired RNA transcription and processing [40] have been reported in the nuclei of aged skeletal muscles (both fiber nuclei and satellite cell nuclei), suggesting perturbations during cellular homeostasis that could lead to muscle atrophy and dysfunction.

In this view, the present paper offers an overview of the current knowledge on the nucleus of myofibers and satellite cells in the aged skeletal muscle, focusing on the potential contribution of nuclear pathway impairment to age-related sarcopenia.

## 2. Structural Organization of the Cell Nucleus

The nucleus is the prominent organelle in eukaryotic cells. Through its envelope, the nucleus physically separates the genome as well as all the machineries involved in gene expression, DNA and RNA synthesis, transcription, and processing from the cytoplasm. The nuclear envelope consists of two membranes, termed the outer and inner membranes, that fuse at the nuclear pore complexes [41]. The nuclear pore complex is a small channel, composed of several proteins (i.e., nucleoporins) that allow for the bidirectional transit (influx and export) of proteins and RNA between the nucleus and the cytoplasm. Far from being simply a passageway, nuclear pores have been shown to be involved in the regulation of gene expression, cell cycle progression, the maintenance of the integrity of the nuclear envelope, and chromosome segregation [42]. Below the inner nuclear membrane, a dense protein network of nuclear lamins (i.e., the nuclear lamina) participates in determining the size, shape, and stiffness of the nucleus [43,44,45], as well as regulating DNA organization and repair, and transcriptional processes [46]. All this is mediated through the direct or indirect interaction of the nuclear lamina with chromatin [47] and transcription factors [48,49]. The nuclear lamina structurally and functionally connects the nucleoskeleton (intranuclear structural proteins, which act as scaffolds in transcriptional regulation and DNA synthesis and transcription) with the cytoskeleton by sensing the cytoskeletal forces and thus mediating the regulation of chromatin organization, gene expression, transcriptional activity as well as biochemical signaling and general cell function and adaptation [50,51,52,53].

Through the nuclear lamina interface, chromatin distributes to the nuclear periphery. Here, condensed chromatin possesses heterochromatin markers that characterize it as transcriptionally silent [54]. Indeed, condensed chromatin organization represses the binding of RNA polymerase [55] and transcription factors [56]. A remodeling of chromatin into decondensed active euchromatin is therefore required during gene transcription [57] to allow for the assembly of the transcription machinery [58]. RNA transcription and processing are dynamic processes in which several key players are involved. The cell nucleus is thus organized in domains, which are the morphological expression of RNA transcription, processing, and cleavage [37], while being the sites of storage and assembly of the factors engaged in these processes [36]. A special mention is given to ribonucleoprotein (RNP)-containing structural constituents occurring in the interchromatin space with a well-defined spatial distribution corresponding to the chronological order of transcriptional and post-transcriptional events, such as, e.g., the perichromatin fibrils (the in situ form of nascent mRNA), located in the perichromatin region in close proximity to the transcribing euchromatin and the perichromatin granules (storage sites of spliced messenger (m)RNA ready to be exported), occurring in both perichromatin and interchromatin regions until the nuclear pore’s entrance is opened [59]. Among the nuclear compartments, the nucleolus is the organelle where rDNA genes are located and the synthesis and maturation of pre-ribosomes take place [60]. Furthermore, the nucleolus has been shown to be a storage and/or passage site for factors involved in functions other than ribosome biogenesis, strongly suggesting its multiple functional roles [61].

Skeletal muscle fibers are a syncytia containing hundreds of nuclei (myonuclei), which are generally considered as post-mitotic nuclei. However, Borowik et al. [62] observed DNA synthesis in the tibialis anterior myonuclei of mice, thus demonstrating that adult myofibers are not completely post-mitotic.

Each myonucleus may potentially perform distinct functions and express different sets of genes; however, specific regions of the myofiber show functional specialization, requiring localized transcripts, for example, at the neuromuscular junction [63,64,65,66] and muscle–tendon connection sites [67]. It has been therefore proposed that modest transcriptional heterogeneity occurs among myonuclei based on their association with anatomically distinct sites as well as the occurrence of stochastic phenomena [68,69]. Regardless, the overall structural organization is maintained.

In the following chapters, we summarize the structural and functional changes that myofiber and satellite cell nuclei undergo during the physiological aging of skeletal muscle fibers.

## 3. The Myonucleus in Aging

Skeletal muscle fibers are a syncytia whose cytoplasm is almost entirely occupied by longitudinally arranged myofibrils. In each myofiber, hundreds of myonuclei are peripherally located and evenly spaced [33,70] beneath the sarcolemma. The number and spatial distribution of myonuclei are crucial for myofiber functionality and as determinants of mammalian skeletal muscle size [71]. Each myonucleus governs a defined volume of the cytoplasmic territory (the myonuclear domain), thus minimizing the extent of the cytosolic transport of gene and protein products [33,72] and efficiently regulating their distribution [73,74]. The spatial location of myonuclei is finely tuned to maximize the distance between them [70] without exceeding the maximum functional capacity of each myonucleus (the physiological “ceiling” [75]) so that sustainable muscle development is maintained for life [71].

Several studies have been performed on the myonuclei of skeletal muscles during aging, but the results have not always been consistent. 

### 3.1. Number, Size, and Shape of Myonuclei

Immunohistochemical analyses revealed an increased amount of myonuclei in the quadriceps femoris [76], tibialis anterior [77] and vastus lateralis [78] muscles of old human subjects as well as in the biceps brachii and quadriceps femoris muscles of aged rats [79], thus supporting the idea of a decrease in cytoplasm volume according to the age-related muscle mass loss. Thus, it has been proposed that the number of myonuclei determines the final size of the muscle, influencing its degree of flexibility [71]; however, an unchanged number of myonuclei was reported in human quadriceps femoris [80] and vastus lateralis muscles [81] as well as in the soleus muscle of old rats [82]. Finally, other authors have observed a decreased number of myonuclei in various murine muscles, which may be due to a decreased and/or less efficient replacement of myonuclei by the satellite cell pool [70,83] or due to myonuclear apoptosis (e.g., [84,85]), although apoptosis has been found to scarcely occur in aged skeletal muscles [79,86]. 

The observed inconsistent results likely depend on the differences in the experimental models, age, muscles, and fiber type studied. In addition, the discrepancies in the results may, at least partially, depend on an erroneous myonuclear count due to the formation of myonuclear aggregates [78] and nuclear chains [83] in aged fibers, as shown by a histochemical analysis of both human vastus lateralis and mouse tibialis anterior muscles, respectively. 

Some studies described alterations in the shape of the myonuclei, which in aged muscle fibers are often irregular (as observed by a histochemical analysis of mouse extensor digitorum longus and tibialis anterior muscles [70,83]), deviating from the round/elliptical shape typical of young fibers (as described in human vastus lateralis muscles [78]) and showing indentations of the nuclear envelope (as observed by transmission electron microscopy in murine quadriceps muscles [87]). Interestingly, the changes in shape and the less ordered distribution of myonuclei have been related to age-dependent alterations in the microtubule network that surrounds each myonucleus [70]. It is assumed that a more or less constant myonuclear domain should be maintained in myofibers [88]. The loss of myonuclei or the reorganization of their spatial positioning due to aging can alter the extent of the myonuclear domain, thus compromising the efficiency of the myonucleus in governing specific cytoplasmic territories. An aging-associated decrease in the size of the myonuclear domain was described in fast type II fibers of the human vastus lateralis muscle, while in slow type I fibers, the myonuclear domain size increased, with a high number of very large myonuclear domains [78]. This suggested a physiological/functional fiber-type-dependent adaptation to the age-related atrophy. Levy et al. [89] suggested that the changes in the spatial distribution of myonuclei affecting the size of the myonuclear domain may impair the effective distribution of gene products as well as protein turnover, leading to a decrease in the concentration of contractile proteins with a reduced ability of myofibers to generate force [90,91]. This would result in skeletal muscle weakness, a typical hallmark of sarcopenia.

### 3.2. Myonuclei as Mechanosensors

Myonuclei also function as cellular mechanosensors [53,92], which makes their optimal spatial organization even more crucial. The nuclear envelope and its nuclear lamina physically separate the nuclear genome from the cytoplasm, and together, they mediate the transmission of cytoplasmic mechanical forces to the nuclear interior, thus promoting changes in the arrangement of chromatin and nuclear domains [93,94,95]. As already recalled, the nuclear lamina is, in fact, involved in the regulation of the nuclear size and shape [43], and affects chromatin architecture [96] and the transcription process [46]. In the quadriceps femoris myofibers of aged mice, Iyer and coworkers [87] described defects in the myonuclear lamina (i.e., the reduced expression and accelerated loss of lamin-β1) and in the nuclear pore complexes (i.e., the reduced number of nuclear pores as well as the decreased expression of Nucleoporin 107 and increased expression of Nucleoporin 93). The authors hypothesized that the nuclear mechanosignaling and the response to mechanical forces may be compromised in aging, thus contributing to sarcopenia. Defects in the nuclear pore complexes and alterations in the nuclear lamina may also be the cause of the age-related inappropriate permeability of the myonucleus, as suggested by increased nuclear influx traffic in the myonuclei of aged skeletal muscle in comparison with their younger counterpart [87]. 

### 3.3. Myonuclear Activity

**Figure 1:**
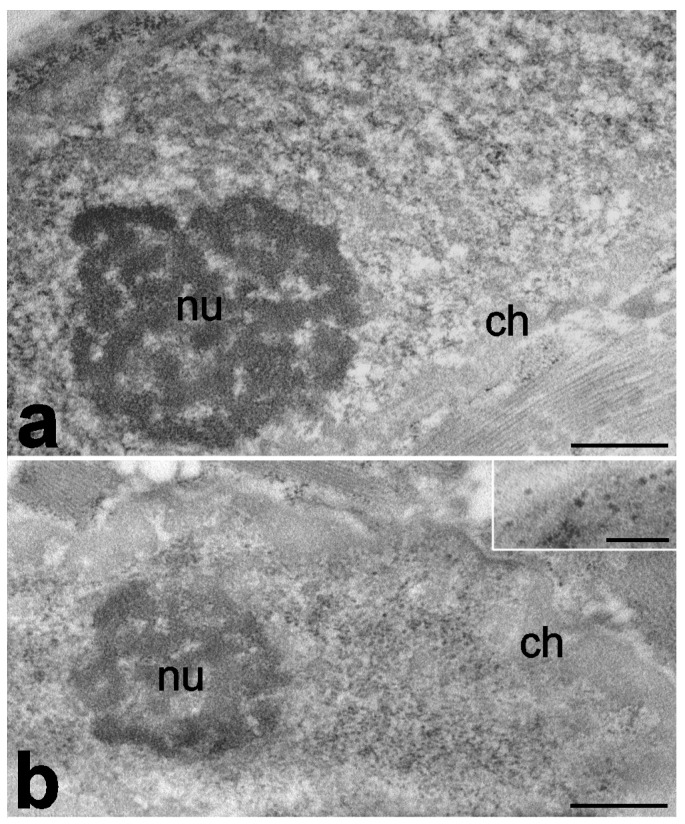
Transmission electron micrographs of myonuclei of quadriceps muscles from young (**a**) and old (**b**) rats. Samples were fixed with paraformaldehyde, embedded in acrylic resin, and stained via ethylenediaminetetraacetic acid (EDTA) method, which reveals RNP-containing nuclear constituents by bleaching the condensed chromatin. In old animals, condensed chromatin (ch) is more abundant, and perichromatin granules (inset) are more numerous than in adult ones, whereas nucleoli (nu) show similar characteristics. Bars: 500 nm; inset: 250 nm [79].

Changes in the nuclear shape and chromatin conformation can directly affect transcriptional regulation [97]. An ultrastructural immunocytochemical analysis of biceps brachii and quadriceps femoris muscles of aged rats showed that myonuclei have a smaller area in comparison to young animals and are characterized by a higher fraction of condensed chromatin, lower number of nucleoplasmic factors involved in mRNA transcription and processing (e.g., RNA polymerase II and splicing factors), and the nuclear accumulation of mRNA cleavage factors and polyadenylated RNA [79] (Figure 1). As a whole, these features suggest an impairment in the production rate and nucleus-to-cytoplasm export of mRNAs. 

Consistently, an increase in condensed chromatin also occurs in the quadriceps femoris muscle of aged mice, along with the relocation of the post-transcriptional splicing regulator, Muscleblind-like protein [98], from its functional intranuclear sites to transcriptionally inactive RNP-containing domains, where it accumulates [99]. It is worth noting that exercise is able to promote the reactivation of mRNA transcription and processing in the myonuclei of the quadriceps and gastrocnemius muscles of aged mice, bringing the amount of transcription, maturation, and cleavage factors back to the values observed in adult animals [100].

### 3.4. Epigenetic Changes in Myonuclei

Epigenetic changes represent the primary hallmark of aging and the dysregulation of DNA methylation (i.e., the addition of a covalent methyl group to the 5′ position of the pyrimidine ring of a cytosine (5-mC)) occurs in aged skeletal muscle at tissue-specific genes [101]. A genome-wide investigation of the DNA methylation status of the vastus lateralis muscle revealed a predominant pattern of hypermethylation in healthy old male humans compared to young individuals. Differentially methylated cytosine–guanine (C–G) pairings (CpG sites) were preferentially located within the gene and in the central and 3′ end regions of the gene while being underrepresented in the promoter region [102]. The hypermethylation of CpG-rich regions, such as gene promoters, typically leads to the reduced ability of the transcriptional apparatus to bind to these regions, effectively suppressing gene expression [103]; therefore, it has been suggested that intragenic DNA methylation may regulate alternative splicing [104], modulate alternative promoter activity [105], or be involved in protective mechanisms [102]. The gastrocnemius muscle of old mice showed the hypermethylation of 762 distinct promoter CpG sites that mapped to one or more of 133 different genes, with an over-representation of genes associated with NAD activity [106], thus explaining the widespread reduction in tricarboxylic acid cycle proteins described in aged human muscle using exploratory proteomics [107]. In the same mice, progressive weighted wheel running (developed by [108]) attenuated the age-associated hypermethylation of promoter region of *Rbm10*, a pleiotropic factor generally dysregulated in aged skeletal muscle [107], and implicated it in alternative splicing [109] and striated muscle hypertrophy [110]. The lower hypermethylation of the *Rbm10* promoter induced by exercise was also confirmed by a high-resolution targeted methylation analysis [106]. An analysis of the CpG islands (i.e., CpG clusters) in aged mouse muscle also revealed the hypomethylation of eight genes, including *Hoxa3* [106], a member of the homeobox (HOX) family of genes targeted by age-associated methylation changes in muscles [111,112]. By using a differential methylation analysis, Turner et al. [111] demonstrated a considerably hypermethylated profile/signature in aged human vastus lateralis and gluteus medius muscles compared with young samples, with an enrichment in methylated CpG sites in the HOX family of genes. 

It is worth considering that the DNA methylation profiles described for skeletal muscles probably reflect the co-presence of other cell types in the tissue besides myofibers. Indeed, although myonuclei represent the main source of DNA in skeletal muscle tissue samples, contamination by non-muscle cells may influence muscle-specific methylation signatures. 

Several CpG sites were differentially methylated (both hyper- and hypomethylated) in the rDNA of aged muscle with the likely implication of ribosome biogenesis [106], a process induced during muscle hypertrophy [113]. The methylated form of the DNA base cytosine, 5-methyl cytosine (5-mC), is an epigenetic regulator of gene transcription involved in chromatin organization and in the protection from illicit recombination events promoting rDNA loss [114,115], while ribonuclease (RNase) A is responsible for the intranuclear RNA degradation and the activation of rDNA transcription [116,117]. In the myonuclei of the rectus femoris muscle of old mice, lower nucleolar amounts of both 5-mC and RNase A were observed when assessed by quantitative ultrastructural immunocytochemistry, suggesting an age-dependent loss of rRNA genes. In the same myonuclei, nuclear actin (i.e., the motor protein regulator of RNA transcription and a marker of intranuclear motility [118]) was observed to decrease, which strongly suggests an impairment in mRNA transcription and/or the nucleus-to-cytoplasm export of both mRNA and ribosomal subunits [119].

The age-related impairment in the biogenesis of pre-ribosomes and their transport to the cytoplasm has also been demonstrated in murine biceps brachii [79], quadriceps [79,100], and gastrocnemius [100] muscles by means of ultrastructural morphometry of the nucleolar components. Consistently, using a biochemical fractionation strategy, Cutler et al. [120] demonstrated an increased amount of ribosomal proteins in the myonuclei isolated from the gastrocnemius and rectus femoris muscles of aged mice, while a decrease in ribosome biogenesis was found in aged skeletal muscle by microarray analysis [121]. Through the proteomic analysis of myonuclei purified from the whole muscles of the gastrocnemius and rectus femoris of aged mice, Cutler et al. [120] also found age-related changes (either an increase or decrease) in proteins involved in transcriptional regulation, transcripts’ processing and transport, and chromatin maintenance, collectively indicating an alteration in these mechanisms due to aging. It must be taken into account that the myonuclear genes and proteins may be underestimated given their small presence in the overall muscle genome and proteome. In addition, the specific isolation of myonuclei from the entire skeletal muscle has always been a challenging task, so several approaches have been optimized for downstream applications (e.g., [122,123,124]). 

A subpopulation of myonuclei with a senescent phenotype has been identified [125] in isolated single myofibers from the extensor digitorum longus muscle of aged mice. Here, it was possible to estimate, by quantitative reverse transcription polymerase chain reaction (RT-qPCR) and RNA in situ hybridization (RNA-ISH), the overexpression of *p21* (also known as *Cdkn1a*), a cyclin-dependent kinase inhibitor that governs the senescence program. By using Gene Ontology and Gene Set Enrichment Analysis assays as well as the analysis of Database of Cell Senescence Genes (CellAge), a significant increase in *p53* signaling and cytokine–cytokine receptor interactions were found, confirming the acquisition of a senescence-like transcriptional profile in aging [125]. Consistent with the senescence profile described in aged mice, a small population of *CDKN1A* (cyclin-dependent kinase inhibitor 1A)/*MYH8* (myosin heavy chain 8)/*COL19A1* (Collagen Type XIX Alpha 1 Chain)/*LRRK2* (leucine-rich repeat kinase 2)-expressing nuclei has been identified in the vastus lateralis muscle of aged humans by single nucleus profiling [126]. High mRNA levels of the senescence-associated genes *p53* and *p21* have also been found in the skeletal muscle of aged mice, monkeys, and humans [127,128,129], although a differentiated analysis of the type of cell source was performed in none of these studies. The single-nucleus profiling of the tibialis anterior muscles of old mice also revealed myonuclear populations characterized by the upregulation of the *Smad3* and *Nr4a3* genes [69], which are implicated in the muscle-aging process [130,131] and metabolic adaptation to exercise [132,133], respectively.

Jing et al. [134] used the RNA sequencing of a single nucleus from the quadriceps femoris muscle of a non-human primate to demonstrate the upregulation of genes associated with the response to muscle inactivity (i.e., the loss of muscle mass and strength typical of aging and muscle disuse) and the downregulation of genes related to the maintenance of muscle structure and function. Among these genes, *FOXO3* (forkhead box protein O3) was found to be downregulated by the integrated analysis of differentially expressed genes associated with aging and the Atlas of Ageing database [134]. *FOXO3* is a pro-longevity gene [135,136,137] whose product acts as a stress sensor and homeostasis regulator [138,139]. *FOXO3* transcripts were downregulated as much as the *FOXO3* target genes, including *AKT3* (AKT serine/threonine kinase 3), *PIK3R1* (phosphatidylinositol 3-kinase regulatory subunit alpha), *FOXO1* (forkhead box protein O1) (all relevant for longevity pathways), and *GPCPD1* (glycerophosphocholine phosphodiesterase 1), *PDLIM3* (PDZ and LIM domain 3), *ARID5B* (AT-rich interaction domain 5B); all these genes are involved in the development of muscle structure or the maintenance of homeostasis [134]. A *FOXO3* deficiency, as assessed in human *FOXO3* knockout myotubes, promoted accelerated aging that led to changes in gene expression similar to those observed in the skeletal muscle of aged monkeys. The co-immunoprecipitation of *FOXO3* with TRF2 (terminal restriction fragment 2, one of the human proteins forming the capping complex directly bound to telomeric DNA) demonstrated that *FOXO3* associates with telomeres in human skeletal muscle, and that this association increases with age (while the TRF2 level decreases). This suggests that *FOXO3* mediates telomere protection even if TRF2 is downregulated [140]. 

### 3.5. Telomeres in Myonuclei

Telomeres are specialized nucleoprotein (TTAGGG repeats) caps located at the ends of eukaryotic chromosomes, protecting their integrity; however, telomeres undergo erosion during each round of cell division, resulting in the loss of telomere length during aging [141,142]. Remarkably, telomere shortening proved to impair tissue renewal [142,143], thus representing a possible contributing factor to sarcopenia, and it is worth noting that physical activity, known to limit sarcopenia (e.g., [144,145]), is able to limit telomere shortening in skeletal muscle [146,147,148]. Ponsot et al. [149] observed that the mean telomere lengths did not significantly shorten in tibialis anterior muscle of elderly human subjects. This finding is consistent with the fact that skeletal muscle fibers have a very long lifespan and contain post-mitotic myonuclei that have undergone a few mitotic divisions before differentiation, thus keeping the telomere length relatively constant and unchanged. Similarly, the detection of telomere restriction fragments by hybridization to a ^32^P-TTAGGG probe did not reveal any alteration in the mean telomere lengths of masseter and biceps muscles of elderly humans in comparison to young counterparts [150]. Moreover, no age-related difference in the mRNA expression of both TRF1 (terminal restriction fragment 1) and TRF2 was highlighted in rat muscles [151]; However, the mRNA expression of telomerase, an enzyme that elongates telomeres, was slightly higher in the same aged muscle [151].

**Figure 2:**
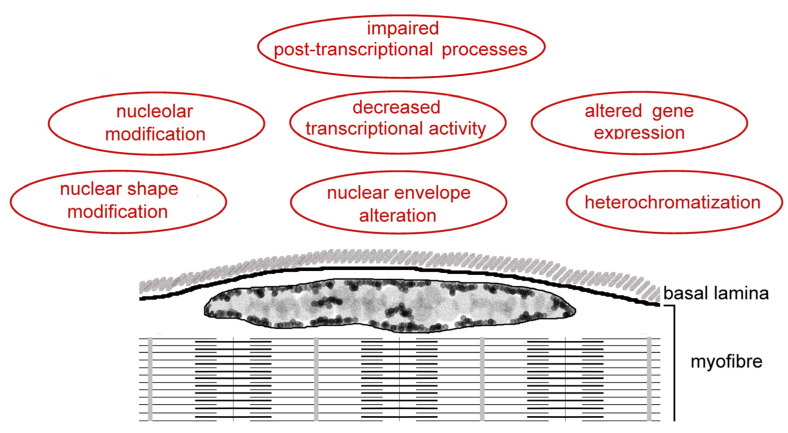
Schematic representation of a skeletal muscle fiber with a myonucleus located just below the sarcolemma. This figure summarizes the age-related alterations in myonuclei described in Section 3.

Figure 2 and Table 1 summarize the findings on myonuclei in the elderly, as described in this chapter.

## 4. The Satellite Cell Nucleus in Aging

Satellite cells are postnatal myogenic stem cells [152] laying between the basal lamina and the sarcolemma of skeletal muscle fibers [153]. They are characterized by a small size with a minimal amount of cytoplasm and organelles, and a single heterochromatic nucleus [154]. In adult muscles, satellite cells are usually in a quiescent state; however, in response to physiological or pathological stimuli (e.g., exercise, mechanical injury, denervation, and muscle dystrophy), they are activated, re-enter the cell cycle, and proliferate, generating myoblasts, and some of the resulting daughter cells return to quiescence to maintain the basal satellite cell population [155,156,157,158,159]. Satellite cell-derived myoblasts may repair existing fibers to compensate for the muscle turnover or fuse together to generate entirely new multinucleated syncytia, thus supporting skeletal muscle plasticity [160,161]. Notably, satellite cells are greatly influenced by the local microenvironment (the so-called satellite cell niche) throughout their activation, proliferation, and differentiation processes, leading to muscle regeneration. Multiple environmental factors (e.g., cytokines, growth factors, free radicals, ion concentration, and mechanical cues) are able to trigger intracellular signaling cascades that ultimately target the satellite cell nucleus and regulate gene expression [162,163]. 

Satellite cells therefore represent a key factor in muscle growth, maintenance, and regeneration, and they have inevitably attracted the attention of scientists as tissue components possibly involved in age-related muscle atrophy. 

### 4.1. Number of Satellite Cells

A reduced number of satellite cells has been sometimes reported in aging muscle, although this finding varies depending on the fiber type and species. This suggests that the functional defects of satellite cells may also contribute to the age-related loss of muscle mass by reducing their response to proliferation and differentiation stimuli (e.g., [83,150,164,165,166,167,168,169,170,171,172,173]). It should be noted that the direct role of stem cells in the maintenance of fiber size during aging has been questioned since sarcopenia proved to be unaffected in satellite-cell-depleted mice [174]. 

### 4.2. Myogenic Potential and Nuclear Activity of Satellite Cells

Myogenesis is regulated by a number of transcription factors, including paired box protein 3 (Pax3), paired box protein 7 (Pax7), myoblast determination protein 1 (MyoD), myogenin, myogenic factor 5 (Myf5), and myogenic factor 6 (Myf6) [175]. Myogenic regulatory transcription factors, typically occurring in satellite cell nuclei, have been widely used as markers of satellite cell presence and activation in aged skeletal muscles, showing heterogeneous results. When the expression levels of Pax7 (a marker of both quiescent and activated satellite cells), MyoD (a marker of activated and proliferating satellite cells), and myogenin (a marker for satellite cell commitment) were assessed by immunohistochemistry in satellite cells of the gastrocnemius and triceps muscles of aged and young rats, only a decrease in Pax7 in aged gastrocnemius muscles was found, suggesting the decline in satellite cell number as a main factor contributing to muscle mass loss rather than a decreased differentiation capability [176]. On the other hand, the RT-PCR analysis of Pax7, MyoD, and Myf5 (another transcription factor involved in the control of satellite cell differentiation [177,178]) in gastrocnemius muscles showed a decrease in the mRNA of all factors in satellite cells of aged mice in comparison to young animals, thus suggesting a decline also in myogenic capability [179]. In addition, it was found that following physical exercise, wingless-related integration site (Wnt)/β-catenin signaling increases the expression of the *Myf5* and *MyoD* genes through the association of the β-catenin·TCF (T-cell factor)·LEF (lymphoid enhancer factor) activator complex with the promoters of these genes in satellite cell nuclei [179].

To understand the reasons for the age-related impairment of skeletal muscle regeneration with age, the Notch signaling pathway, which plays a key role in satellite cell activation and adult muscle regeneration [180], was analyzed in satellite cells isolated from mice hindlimb muscles by combining histochemical and molecular techniques. The results demonstrated that the reduced proliferation and differentiation capability of satellite cells observed in old animals was accompanied by the insufficient upregulation of the Notch ligand Delta and, in turn, the diminished activation of Notch [181].

Androgens, and in particular testosterone, play a key role in increasing skeletal muscle mass and strength [182] by stimulating satellite cells [183]. Accordingly, satellite cells have been found to express androgen receptors [184]. Recently, by combining ex vivo and in vitro studies, Di Donato et al. [185] demonstrated a lower level of androgen receptors in biopsies of the gluteus medius or vastus medialis muscles of old vs. young human subjects, as well as an androgen-induced nuclear translocation of an androgen receptor in cultured murine myoblasts. This suggests that the decrease in androgen level with aging may lead to a functional decline in satellite cells by reducing nuclear signaling. 

**Figure 3:**
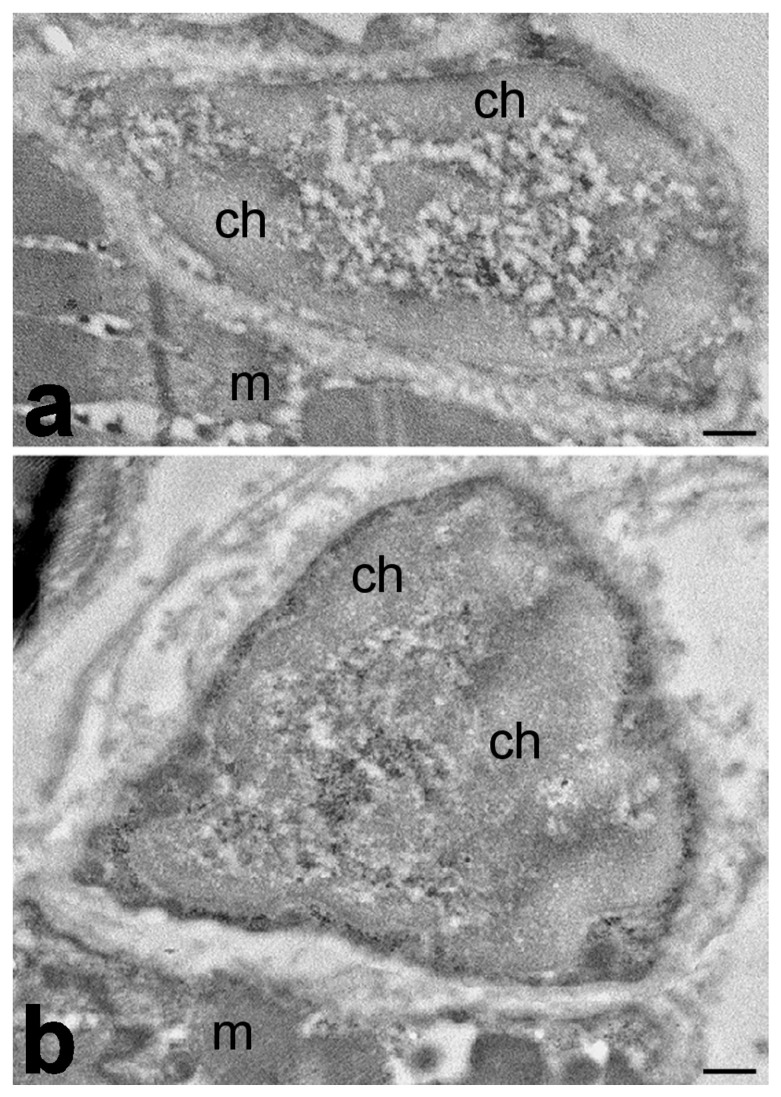
Transmission electron micrographs of satellite cells of quadriceps muscles from young (**a**) and old (**b**) rats. Samples were fixed with paraformaldehyde, embedded in acrylic resin, and stained via EDTA method, which reveals RNP-containing nuclear constituents by bleaching the condensed chromatin. No evident ultrastructural modification of nuclear structural constituents occurs in satellite cell nuclei of young vs. old animals. Condensed chromatin (ch); myofiber (m). Bars: 500 nm.

The fine morpho-functional features of satellite cell nuclei have been investigated by transmission electron microscopy and ultrastructural cytochemistry in the biceps brachii and quadriceps femoris muscles of old rats, demonstrating that although the structural features of the nuclear components did not change in comparison to young animals, a significant reduction in pre-mRNA splicing, cleavage, and intranuclear transport occurs during aging (Figure 3). This impairment of nuclear post-transcriptional activities suggests that the responsiveness of satellite cells to muscle damage could be hampered in the elderly [186].

Similar observations have been reported in the satellite cell nuclei of quadriceps the femoris and gastrocnemius muscles of old mice, where a decrease in transcription activity was also found in comparison to young animals [100]. Interestingly, when old mice underwent adapted physical exercise, their satellite cell nuclei showed only a partial restoration of the post-transcriptional pre-mRNA pathways, demonstrating a limited—although still present—capability to respond to mechanical stimuli. This supports the hypothesis that age-related alterations of transcriptional and post-transcriptional pathways in satellite cell nuclei may lead to a reduced activation and differentiation capability, thus contributing to the loss of muscle mass in the elderly. Accordingly, the myogenic potential of satellite cells isolated from the quadriceps femoris muscles of old mice proved to be significantly reduced in comparison to young animals, with the satellite-cell-derived myoblasts showing nuclei with reduced amount of transcriptional and post-transcriptional factors [187]. Again, when old mice underwent physical exercise, post-transcriptional factors were partially restored in myoblast nuclei, and an improvement in the in vitro differentiation capability was observed [187].

### 4.3. Telomeres in Satellite Cell Nuclei

Special attention has been given to telomeres. Satellite cells are stem cells that remain in a quiescent state for a long time and therefore undergoing only a few mitotic divisions during their lifespan. Consistently, satellite cells isolated from the tibialis anterior, quadriceps and gastrocnemius muscles of aged mice and analyzed by quantitative fluorescence in situ hybridization showed no significant shortening of their telomeres in comparison to young animals, suggesting that telomere length is stable in aging murine satellite cell nuclei [188]. In humans, the telomere length of satellite cells isolated from the quadriceps femoris muscle was measured in infants (5 days and 5 months), and adult and old subjects, finding longer telomeres in infants but similar lengths in young (26–31 years) and elderly (62–81 years) individuals [189]. Accordingly, no change in telomere length was found in satellite cells from the vastus lateralis and gluteus medius muscles [190] and in various skeletal muscles [191] of young and old humans. Satellite cells isolated from vastus lateralis biopsies of human volunteers were used to assess telomere length and damage by immunocytochemistry, in situ hybridization, and fluorescence microscopy, again demonstrating no change between young and old subjects. On the other hand, dysfunctional telomeres were found to increase in satellite cell nuclei from old subjects, without any resumption after physical exercise [192]; therefore, damaged rather than shortened telomeres may represent a contributing factor to the decline in the regenerating capability of satellite cells in old muscles. This hypothesis is supported by experimental findings on telomerase, an enzyme that maintains telomere length by adding guanine-rich repeats [193]. Telomerase activity was assessed by enzyme-linked immunosorbent assay in satellite cells isolated from the gastrocnemius, quadriceps, and hamstring muscles of aged mice. The results demonstrated that old satellite cells maintain their telomerase activity at a level similar to that of young animals and generate myofibers with no telomere shortening [194], thus excluding the involvement of telomerase dysfunction in the sarcopenic drive; however, it should be taken in consideration that mice used in research laboratories have longer telomeres and higher levels of constitutive telomerase activity than humans. Consequently, human and mouse satellite cells could age differently [195,196].

### 4.4. Epigenetic Changes in Satellite Cell Nuclei

Satellite cells, as adult stem cells, are characterized by a very long lifetime and an extremely low turnover rate, undergoing both replicative aging during the proliferative phase (due to, e.g., telomere damage) and chronological aging during quiescence (mainly due to the accumulation of damaged macromolecules) [197]. When the effect of chronological aging was investigated in satellite cells isolated from the limb muscles of old mice by gene expression microarray analysis, a decreased transcription rate of histone genes and a consequent accumulation of repressed chromatin domains were found in comparison to young animals, suggesting that these epigenetic changes may lead to a functional decline in satellite cells with age [198], thus contributing to the age-associated impairment in muscle regeneration. An aberrant epigenetic stress response has been also reported in satellite cells isolated from the hindlimb muscles of old mice [199]. In particular, higher levels of repressive markers and lower levels of histone modifications typically enriched on active genes were observed in quiescent satellite cells of aged mice compared to young animals, while in activated satellite cells of old mice, an increase in chromatin decompaction occurred, impairing myogenic capability, decreasing cell proliferation, and increasing the apoptotic rate. These effects are due to the upregulation of the *Hoxa9* gene (both at the mRNA and protein level) in the satellite cell nuclei of old mice. The overexpression of *Hoxa9* (homeobox A9), one of the master regulators of embryonic development [200] therefore limits satellite cell self-renewal and muscle regeneration in old mice, likely contributing to sarcopenia. On the other hand, *Hoxa9* represents also a potential therapeutic target since its deletion is sufficient to revert impairments in satellite cell activation.

In a recent study [201], satellite cells isolated from the hindlimb muscles of young, aged, and geriatric mice were analyzed for their transcriptional changes and for 3D genome organization. The results demonstrated distinct patterns in the three animal groups, with the upregulation of transcripts related to cell adhesion, of inflammatory and immune responses in aged vs. young mice, and of transcripts related to cell surface and extracellular space in geriatric vs. aged animals. Similarly, 3D genome organization showed distinct patterns in the three animal groups, with increased long-range interactions in geriatric (i.e., 28–32 months of age) vs. aged (23–24 months) and young (2 months) mice, increased interaction strength between active euchromatin compartments in aged vs. young mice, and decreased interaction strength between inactive heterochromatin compartments in geriatric vs. aged mice. In addition, the interaction strength of chromatin loops increased in aged and geriatric mice vs. young ones. Although no direct correlation was observed between genome compartmentalization and gene expression changes, this pioneer study suggests that satellite cell nuclear dysfunction during aging may be ascribed to changes in transcriptional networks and chromatin states, thus offering a promising key to better understand sarcopenia and develop novel therapeutic approaches. Accordingly, modifications in the transcriptome have been found in satellite cells isolated from the hindlimb muscles of old mice [202], evidencing, e.g., an increased expression of stress response and antioxidant genes and a decreased expression of genes involving extracellular matrix interactions. These transcriptome changes were correlated with changes at the level of chromatin accessibility and DNA methylation. Interestingly, many (but not all) of the observed age-related transcriptome alterations in satellite cells were niche-dependent since they were reversed after transplantation in young animals.

It is well known that DNA methylation undergoes dysregulation during aging (recent reviews in [203,204]), gaining or losing methylation at different genomic loci, a phenomenon called methylation drift [205]. Vaidya et al. [206] investigated this phenomenon by comparing stem and differentiated cells of various tissues of young and old mice and demonstrated that, since DNA methylation/demethylation occurs during DNA replication, the age-related methylation drift is tissue-specific and linked with stem cell division. Consequently, a limited (although significant) methylation drift was found in skeletal muscle nuclei (both satellite cell nuclei and myonuclei) of old versus young mice in comparison to high-proliferating tissues because of the very low rates of muscle satellite cell division. An increased level of DNA methylation related to genes involved in myogenic differentiation and quiescence was observed also in satellite cells from the quadriceps muscles of old vs. young human subjects, suggesting an impairment of the self-renewal capacity [207]. Similarly, DNA methylation was found to increase in satellite cells isolated from biopsies of vastus lateralis or gluteus medius muscles of old vs. young human volunteers, mainly consisting of the hypermethylation of genes involved in developmental processes and calcium signaling [111]. 

Interestingly, myoblasts derived from satellite cells isolated from vastus lateralis muscle biopsies of old volunteers showed DNA methylation changes in genes associated with tissue homeostasis, the regulation of stress activation, and the insulin and 5′ AMP-activated protein kinase (AMPK) signaling pathways, suggesting that such changes may contribute to the dysfunction of these pathways, leading to insulin resistance [208], a factor involved in the pathogenesis of sarcopenia [209].

The epigenetic regulation of chromatin condensation is essential to maintain genome integrity and function. In particular, the trimethylation of histone 3 lysine 9 (H3K9me3) is required for the transcriptional silencing of many genes [210]. By focusing on *Hairless* (*Hr*), a gene expressed in quiescent satellite cells and downregulated during activation, Liu et al. [211] demonstrated that the loss of *Hr* expression in satellite cells isolated from the hindlimb muscles of old mice leads to a reduction in H3K9me3 levels and an increase in DNA susceptibility to genotoxic stress, raising the satellite cell death rate after activation and accelerating their depletion due to aging. 

By investigating satellite cells obtained from the murine tibialis anterior muscle of young and old mice, Sahu et al. [212] demonstrated that in old injured muscles the expression of the aging suppressor gene *Klotho*, whose deficiency is involved in the onset of sarcopenia, is prevented due to the loss of the demethylation of its promoter (which normally occurs in young injured muscles), suggesting that the epigenetic control of *Klotho* is lost with aging, thus hampering muscle regeneration.

**Figure 4:**
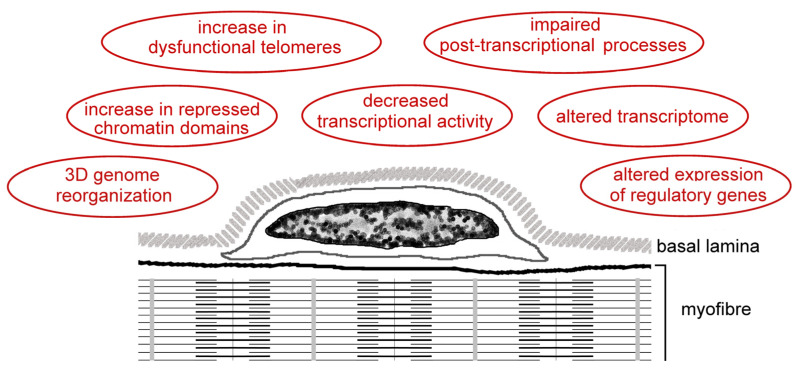
Schematic representation of a satellite cell located between the myofiber sarcolemma and the basal lamina. This figure summarizes the age-related alterations of satellite cell nuclei described in Section 4.

Figure 4 and Table 2 summarize the findings on satellite cell nuclei in the elderly, as described in this chapter.

## 5. Conclusions

The scientific literature on the cell nuclei of aged skeletal muscles is undoubtedly heterogeneous for experimental models, methodology, and techniques, thus providing fragmentary and sometimes inconsistent results; however, taken together, the findings give a complex picture of the morpho-functional modifications of myonuclei and satellite cell nuclei due to aging, which may respectively influence the size and function of the myonuclear domains and stem cell activation and differentiation, thus accounting for the age-related impairments in muscle homeostasis, function, and regeneration. Based on the current knowledge, it is hard to establish which alterations act as the primary causes of age-related muscle decline and which ones are driven by other aging-associated modifications and should be thus considered as secondary causes. Regardless, identifying the nuclear dysfunctions involved in skeletal muscle aging is crucial to understand the molecular and metabolic events leading to the loss of muscle mass and function, and may provide a key to open the doors to therapeutic treatments aimed at limiting or even preventing the sarcopenic process. 

## Figures and Tables

**Table 1:** This table summarizes the reported findings on myonuclei in aging.

Analyzed Features	Species	Main Findings	References
Number of myonuclei	human	Increases with aging	[76,77,78]
	human	No change with aging	[80,81]
	rat	Increases with aging	[79]
	rat	No change with aging	[82]
	mouse	Decreases with aging	[70,83]
Myonuclear spatial organization	human	Changes with aging	[78]
	mouse	Changes with aging	[83]
Myonuclear domain	human	Changes with aging (increases or decreases depending on the fiber type)	[78]
Myonuclear shape	human	Changes with aging	[78]
	mouse	Changes with aging	[70,83,87]
Myonuclear envelope	mouse	Changes with aging	[87]
Myonuclear area	rat	Decreases with aging	[79]
Condensed chromatin	rat	Increases in amount with aging	[79]
	mouse	Increases in amount with aging	[98]
Nuclear activity	rat	Decreased mRNA production with aging	[79]
	mouse	Decreased mRNA production with aging	[100,119]
	mouse	Impairment of mRNA transport with aging	[119]
	mouse	Nucleolar changes with aging	[119]
	mouse	Change in pre-ribosome biogenesis and transport with aging	[79,100,120,121]
	mouse	Changes in transcriptional and post-transcriptional processes with aging	[120]
Gene expression	mouse	Changes with aging	[69,125]
	non-human primate	Changes with aging	[134]
	human	Changes with aging	[126]
Epigenetic changes	human	Changes with aging	[102]
	mouse	Changes with aging	[111]
Telomeres	human	No change with aging	[149,150,151]

**Table 2:** This table summarizes the reported findings on satellite cell nuclei in aging.

Analyzed Features	Species	Main Findings	References
Number of satellite cells	human	No changes with aging	[164,165]
	human	Decreases with aging	[150,166,168,171]
	rat	No changes with aging	[167,170]
	rat	Decreases with aging	[169]
	mouse	Decreases with aging	[83,172,173]
Myogenic potential	rat	No changes with aging	[176]
	mouse	Decreases with aging but increase after physical exercise	[179,187]
	mouse	Decreased Notch signaling with aging	[181]
Nuclear activity	rat	Decreased post-transcriptional activities with aging	[186]
	mouse	Decreased transcriptional and post-transcriptional activities with aging but increases after physical exercise	[100]
Telomeres	human	No changes in telomere length with aging	[189,190,191,192]
	mouse	No changes in telomere length with aging	[188]
	human	Increases in dysfunctional telomeres with aging	[192]
	mouse	No changes in telomerase activity with aging	[194]
Epigenetic changes	mouse	Decreased transcription of histone genes with aging	[198]
	mouse	Overexpression of *Hoxa9* gene with aging	[199]
	mouse	Changes in genome compartmentalization with aging	[201]
	mouse	Changes in transcriptome, chromatin accessibility and DNA methylation with aging	[202]
	mouse	Increased DNA methylation with aging	[206]
	human	Increased DNA methylation with aging	[111,207,208]
	mouse	Decreased expression of *Hairless* gene with aging	[211]
	mouse	Decreased expression of *Klotho* gene with aging	[212]

## Data Availability

The data presented in this study are available upon request from the corresponding author.
